# Study of multiparameter respiratory pattern complexity in surgical critically ill patients during weaning trials

**DOI:** 10.1186/1472-6793-11-2

**Published:** 2011-01-21

**Authors:** Vasilios E Papaioannou, Ioanna G Chouvarda, Nikos K Maglaveras, Ioannis A Pneumatikos

**Affiliations:** 1 Alexandroupolis University Hospital, Intensive Care Unit, Democritus University of Thrace, Dragana 68100, Greece; 2 Laboratory of Medical Informatics, School of Medicine, Aristotle University of Thessaloniki, 54124, Greece

## Abstract

**Background:**

Separation from mechanical ventilation is a difficult task, whereas conventional predictive indices have not been proven accurate enough, so far. A few studies have explored changes of breathing pattern variability for weaning outcome prediction, with conflicting results. In this study, we tried to assess respiratory complexity during weaning trials, using different non-linear methods derived from theory of complex systems, in a cohort of surgical critically ill patients.

**Results:**

Thirty two patients were enrolled in the study. There were 22 who passed and 10 who failed a weaning trial. Tidal volume and mean inspiratory flow were analyzed for 10 minutes during two phases: 1. pressure support (PS) ventilation (15-20 cm H_2_O) and 2. weaning trials with PS: 5 cm H_2_O. Sample entropy (SampEn), detrended fluctuation analysis (DFA) exponent, fractal dimension (FD) and largest lyapunov exponents (LLE) of the two respiratory parameters were computed in all patients and during the two phases of PS. Weaning failure patients exhibited significantly decreased respiratory pattern complexity, reflected in reduced sample entropy and lyapunov exponents and increased DFA exponents of respiratory flow time series, compared to weaning success subjects (p < 0.001). In addition, their changes were opposite between the two phases of the weaning trials. A new model including rapid shallow breathing index (RSBI), its product with airway occlusion pressure at 0.1 sec (P_0.1_), SampEn and LLE predicted better weaning outcome compared with RSBI, P_0.1 _and RSBI* P_0.1 _(conventional model, R^2 ^= 0.874 vs 0.643, p < 0.001). Areas under the curve were 0.916 vs 0.831, respectively (p < 0.05).

**Conclusions:**

We suggest that complexity analysis of respiratory signals can assess inherent breathing pattern dynamics and has increased prognostic impact upon weaning outcome in surgical patients.

## Background

Several indices have been studied for estimation of weaning readiness [1-4]. However, their prognostic value has demonstrated modest accuracy so far, whereas two consensus conferences on weaning did not recommend their routine application in clinical practice and proposed decision-making based on clinical criteria of improvement [3,5].

Recognition that physiologic time series contain hidden information related to an extraordinary complexity that characterizes physiologic systems, has led to the investigation of new techniques from statistical physics for the study of living organisms [6]. Through those techniques different 'physiomarkers' can be estimated, which include variability and complexity indices of different biological signals. Only a few studies have explored indices derived from breathing pattern variability analysis for the estimation of weaning readiness [7-10]. However, different weaning protocols were implemented in heterogeneous groups of patients, using only one and different from each other method for the assessment of breathing dynamics, with conflicting results.

In particular, one study that included medical patients found increased variability and complexity of various ventilatory parameters in those with weaning failure. Two other studies recruited subjects who underwent cardiac and abdominal surgery and found contradictory results in terms of respiratory complexity during weaning trials. Finally, another research group studied a mixed group of patients and showed increased respiratory variability in those who managed to separate from the ventilator. In conclusion, none of the above studies used a combination of different methods for the assessment of complex dynamics of respiratory signals; something that could have increased diagnostic accuracy of such approach.

Variability analysis is not only observing over a longer period of time but much more watching from a different perspective (i.e., how much and why the values are deriving from the mean) [11]. Moreover, it can provide continuous and real time information at any point of the different weaning phases. Coefficients of variation (CVs), spectral and autocorrelation analyses of different respiratory signals are called linear methods and have been implemented for assessing breathing pattern variability and predicting weaning readiness in different groups of mechanically ventilated patients. However, their application supposes stationary time series behaviour, meaning stability of statistical properties of signals along time [11]. Furthermore, they present insensitivity to the orderliness of data and lack the ability of describing system inherent dynamics. For instance, a time series can be very variable but not very complex (oscillation). Conversely, a time series can be less variable but highly complex. For the above reasons, nonlinear methods may better describe nonstationary and nonlinear (continuous and often unpredictable cross-talk between systems' components) properties of a signal [6,11,12].

In the present study and contrary to those that were mentioned previously, we tried to investigate respiratory pattern dynamics using a 'toolkit' of nonlinear methods, in a homogeneous group of surgical critically ill patients during weaning from mechanical ventilation. We wanted to test the hypothesis that reduced respiratory complexity might discriminate weaning failure or success groups. In addition, we examined whether these domains of measurements and their change during weaning trials can predict weaning outcome and therefore identify a unique value of such analysis.

## Methods

### Setting and studying population

This study was performed in a mixed 12-bed Intensive Care Unit (ICU) in the University hospital of Alexandroupolis, Greece, after approval by local Scientific and Ethics Committee. A total of 32 consecutive patients admitted to the ICU from September 2009 to February 2010 who underwent major abdominal surgery [scheduled 18 (56%), urgent 14 (44%)], with a mean Acute Physiology and Chronic Health Evaluation (APACHE) II score upon admission 18.3 (standard deviation: 6.7), were enrolled. There were 25 men and 7 women, with a mean age of 66.4 (SD: 7.9) years.

The whole studying population was divided into successful (S, n = 22) and unsuccessful (U, n = 10) groups according to the weaning outcome. Briefly, the two groups included those who were successfully or unsuccessfully extubated and remained free from invasive or non-invasive ventilation for over 48 hours, respectively. All patients enrolled in the study received mechanical ventilation (model Evita 2 *Dura*, Dräger, Germany) for at least 48 hours and when they met the recommended weaning criteria [3], they underwent their first spontaneous breathing trial (SBT) using low pressure support ventilation (PSV) [4]. Those with cardiac arrhythmias, neurological diseases or pre-medication with cardio-vascular drugs were excluded from the study. Moreover, none from our patients suffered from chronic obstructive pulmonary disease (COPD), minimizing possible effects of chronic hypercapnia on the respiratory centers control.

### Weaning protocol

All patients were under synchronized intermittent mechanical ventilation (SIMV) before the weaning trials, whereas none of them required administration of neuromuscular blocking agents. Patients were ventilated with pressure support (PS) mode for 30 minutes, whereas the pressure level setting was between 15 and 20 cm H_2_O to maintain a tidal volume (V_T_) of approximately 8-10 ml/Kg (stage 1, high support-H). Positive end-expiratory pressure (PEEP) was 5 cm H_2_O, fraction of inspired oxygen concentration was 40% and pressure triggering sensitivity was set on -2 cm H_2_O. Sedatives and opioid analgesics were discontinued in all patients, 24 hours prior to the study, whereas non-steroid anti-inflammatory agents were used occasionally as pain relievers. At the end of this stage, minute ventilation (MV), respiratory rate (RR), V_T_, heart rate (HR) and blood gases were measured in all patients and since they met the weaning criteria, the ventilator mode was switched to 5 cm H_2_O PS plus 5 cm H_2_O PEEP and the other settings remained the same (stage 2, low support-L) for other 30 minutes. When patients completed the 30-min SBT with low PS they were either extubated and considered as weaning success group or were reconnected to the ventilator and considered as weaning failure group (2 of them required reinstitution of mechanical ventilation within 24 hours after extubation and 8 after the performance of weaning trials) [3]. All subjects were kept in semisitting position and left undisturbed throughout the study.

### Respiratory signals acquisition

Data on tidal volume, respiratory rate, minute ventilation and instantaneous ventilatory flow were extracted from the ventilator via a RS232 interface connected to a computer with a Medibus cable, using the software *VentView*^*R *^*2.n (Dräger Medical AG & Co, Lübeck, Germany)*. The signals were not filtered. They were digitized at a 100-Hz sampling rate (PowerLab/4SP, ADInstruments, Castle Hill, Australia), recorded and subsequently analyzed in an HP Pavilion 6181, 2GHz PC. Because oversampling can introduce co-linearities in the signals, the data were subsampled at 5 Hz. Within each 30-min interval and after 10 minutes in each stage (H & L), a stable 10 min time series of V_T _and mean inspiratory flow (V_T_/inspiratory time ratio) that was artefact free was calculated, on a breath-to-breath basis after digital integration of the flow signal. Moreover, episodes of tracheal suctioning, sights or cough were event-marked by the principal investigator and subsequently removed from the respiratory time series, before analysis. Rapid shallow breathing index (RSBI, breaths/min/lt), airway occlusion pressure at 0.1 sec (P_0.1, _cmH_2_O) and their product (RSBI*P_0.1_) were also calculated during SBT [13,14]. Respiratory signals were analyzed off-line by someone blind to weaning trials outcome, according to open-source software from the website http://www.physionet.org, using a computer package (Matlab V.6.5, R13, MathWorks Inc, Natick, MA, USA) [12].

**Table 1:** Respiratory data of the whole study population

Variables	Successful group (S)	Unsuccessful group (U)	p value
	(n = 22)	(n = 10)	
Minute ventilation (MV), L	12.33 (11.21-16.74)	12.95 (11.78-13.2)	NS
RR (respiratory rate) breaths/min	16 (15-18)	17 (15-19)	NS
HR (heart rate) beats/min	82 (77-86)	83 (82-90)	NS
Tidal volume (V_T _) ml	676.2 (543.4-742.2)	665 (583.4-834.8)	NS
pH	7.41 (7.37-7.46)	7.39 (7.37-7.44)	NS
PaCO_2 _mmHg	41.23 (36.2-45.6)	42.65 (38.3-46.8)	NS_2aCOl_
PaO_2 _mmHg	132.3 (98.4-156.7)	131.84 (94.84-148.2)	NS
Male/female (Number)	17/5	8/2	<0.05
Age (years)	65.7 (58-72.3)	67.56 (56.2-74.45)	NS
APACHE II score	16.4 (13.23-19.34)	21.26 (18.76-24.7)	<0.05
Weight (Kg)	76.75 (64.3-82.54)	75.84 (63.2-87.2)	NS
Time of ventilatory support before			
weaning trial (hours)	133.6 (112.5-157.3)	175.6 (142.6-214.2)	<0.05

Respiratory parameters, blood gases and demographic features of the whole study population (n = 32), according to weaning outcome, measured before weaning trials. Data are presented as median (10%-90% percentiles).

### Time series analysis

#### Detrended fluctuation analysis (DFA)

DFA quantifies intrinsic fractal-like (self-similar) correlation properties of dynamic systems, whose basic features is scale invariance, meaning that the same features repeat themselves on different measurement scales [6,12]. The mean inspiratory flow and V_T _interval data after integration were divided into windows of the same size n and subsequently, analysed in relation to a local trend in each window. This procedure was repeated for all different windows. The variability is depicted on a log-log scale as a function of different sizes of windows in a form of linear slope (or self-similar parameter) and characterises the fractal-like correlation properties of the signal. DFA permits the detection of long-range correlations within a time series and has already been applied for assessing fractal properties in highly complex cardiovascular signals [12,15]. Values higher than 1 and towards 1.5 tend to reflect a more periodic and predictable in its evolution time series whereas values lower than 1 and approaching 0.5 characterize a random-like process. For DFA estimation, we used available software from physionet (http://www.physionet.org).

### Sample Entropy (SampEn)

Approximate entropy (ApEn) was introduced by Pincus as a quantification of regularity in data and compares each group of consecutive measurements over a predefined time window to every other group of measurements of the same time length. ApEn is a measure of the likelihood that patterns recur over specified time intervals. Regular signals are expected to have low ApEn, while complex ones take on higher ApEn values [16,17]. Due to ApEn's dependence on the record length an alternative statistic named sample entropy (SampEn) was introduced by Richmann and Moorman [18] with the benefit of reduced computational load.

Sample entropy that represents the negative natural logarithm of the conditional probability that two sequences similar for m points remain similar at the next point with a tolerance r, where self-matches are not included [18], was calculated for flow and V_T _time series. For entropy analysis, different values of parameters (m, r, N) are used for calculations. The N is the length of the time series. The parameter r that is the tolerance for accepting matches, is set between 15-25% of standard deviation (SD) of the time series, after normalization (SD = 1). The parameter m (embedding dimension) is the length of sequences to be compared and its values vary between 1 and 2 for data length ranging from 100 to 5000 data points [16]. In our analysis, we computed SampEn assigning the values of 2 for m and 0.15 for r, according to criteria published elsewhere, in order to minimize the maximum of the relative errors of SampEn and of the conditional probability estimate [19], using software available from physionet (http://www.physionet.org).

### Fractal dimension (FD)

The fractal dimension is another method of quantifying fractal properties of a time series. In this study, FD was estimated in Matlab by use of Higuchi method, which seems to provide more accurate results and incorporates a fast algorithm that requires only short time intervals [20]. FD is based on a measure of length L(k) of a time series, computed at different scales, by using a segment of k samples as a unit in each scale. The value of FD is calculated by a least-squares linear best-fitting procedure as the angular coefficient of the linear regression of the log-log graph of the mean of k values Lm(k) for m = 1,2,3...k, with k being an interval time. The length Lm(k) originating from time m is calculated as the normalized sum of absolute differences between the values of point pairs that are 'k samples distant' and the length of curve of the time interval k, L(k) is calculated as the mean of the k values Lm(k). If the L(k) relates to the scale used (k) linearly in a log-log plot with slope FD, then the curve is said to show fractal dimension. High FD values reflect a high irregularity of the time series and an estimate of the scale-independent complexity of the underlying system (over space or time).

### Largest Lyapunov exponents (LLE )

Complex systems are considered sensitive to initial conditions and exhibit an exponential divergence in the phase space, which describes in a 3-dimensional axis their different states. Estimation of Lyapunov spectrum and largest Lyapunov exponents (LLE) can assess sensitivity to initial conditions. Briefly, if we consider two points in adjacent trajectories-states of the phase space with a distance between them d(0), after time t the average divergence (separation) will be:

(1) $$d\left(t\right)=d\left(0\right)*e^{LLE*(i}{}^{\Delta t)}$$

whereas LLE is the largest Lyapunov exponent. In this study, we computed LLE of mean inspiratory flow and tidal volume signals, using the algorithm proposed by Rosenstein in Matlab, which seems to be useful, particularly in small data sets [21]. Values higher than 0 reflect an unstable and unpredictable system, where nearby points will diverge to any arbitrary separation. Increased LLEs reflect increased sensitivity to initial conditions and characterize unpredictable variations, whereas low values indicate regularity [21].

Finally, three-dimensional distributions of different respiratory signals and phase spaces of mean inspiratory flow and MV, which describe all the possible states (trajectories) of a system, were determined. Briefly, different values of x(i) were plotted against the following ones in tree-dimensional axes: x, x+t and x+2t in Matlab, giving rise to the phase portrait of the signal. This graph is a complicated set of nonrepeating patterns in case of complex systems, whereas in periodic ones resemble a simple closed loop.

### Statistical analysis

Data are presented as median values with 10^th ^and 90^th ^percentiles. Weaning success (S) and failure groups (U) were compared with the nonparametric Mann-Whitney test for continuous variables and the chi-square test for dichotomous variables, whereas different respiratory complexity indices over the 2 phases of PSV were compared with a Wilcoxon paired test. Spearman's ρ was computed for estimating relationships between all variables before and after the SBT and duration of ventilation. A stepwise multiple regression analysis was performed in order to test whether new indices add prognostic value to existing ones and finally, for building a new prognostic model. Moreover, for assessing prognostic accuracy upon weaning outcome of conventional (or model 1 that included RSBI, P_0.1 _and RSBI* P_0.1_) versus new studied indices, a 40-fold cross validation procedure was followed to assess each model's efficiency, using available software from Matlab. In each try, 75% of the dataset was chosen as training set and the remaining 25% as testing set. The regression model was trained with the training set to separate between the two classes. Bootstrapping (500 times) was applied in each try, to ensure more robust estimation of regression parameters, due to small sample size. The bootstrapping procedure involves the repetition of the experiment, each time with a slightly variant dataset produced by the replacement of a dataset sample with another existing one. A distribution is produced for each estimated variable, and the mean value is then used as a robust estimator of the variable in focuses, in this case the regression parameters. In each try, the area under the curve (AUC) and standard error were calculated, along with the best threshold for class separation, and the best sensitivity-specificity pair (in terms of receiver operating characteristic curve-ROC) [22]. All other tests were performed with SPSS Software Version 13.0 (SPSS Inc, Chicago III), whereas values of p < 0.05 were considered to be significant.

## Results

The respiratory parameters, blood gases and demographic data did not differ between the 2 groups before the performance of the weaning trials (Table 1). In addition, mean APACHE II score upon admission and duration of ventilation before the start of SBTs were significantly higher in group U than in group S. Heart and respiratory rate at the end of SBT (phase L) in group U was significantly increased compared with group S [118 (83-132) vs 98 (78-113) and 35 (28-43) vs 28 (22-35), p < 0.001, respectively]. In both groups, HR and RR between the two phases showed significant increase. Correlations between all complexity indices with duration of ventilation were found to be insignificant.

**Figure 1:**
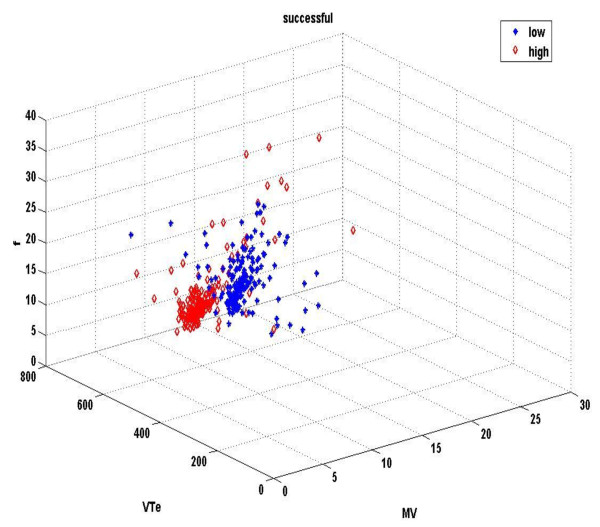
**Three-dimensional distribution of different respiratory signals in a weaning success patient**. Three-dimensional distribution of minute ventilation (MV, lt/min), tidal volume (V_T_, ml) and respiratory rate (RR, breaths/min) of a weaning success patient. Red dots represent data during high PS ventilation, whereas blue dots represent data during the performance of a SBT. Graphics were created in Matlab.

**Figure 2:**
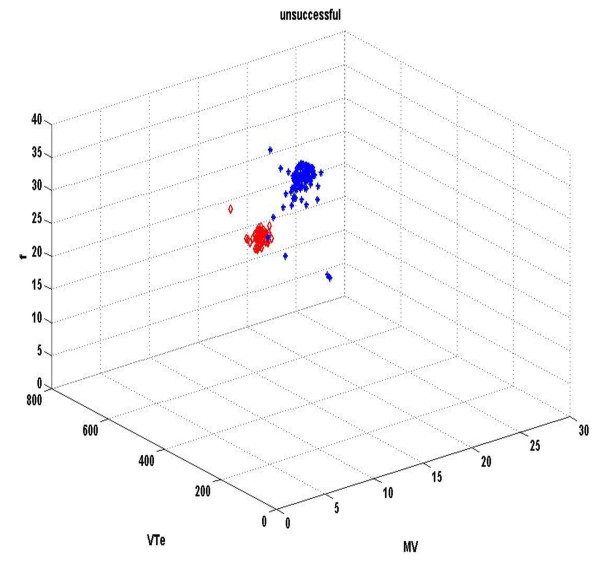
**Three-dimensional distribution of different respiratory signals in a weaning failure patient**. Three-dimensional distribution of MV, V_T _and RR of a weaning failure patient. Red dots represent data during high PS ventilation, whereas blue dots represent data during the performance of a SBT. Dispersion of data seems decreased compared with figure 1, reflecting reduced variability of measured parameters. Moreover, respiratory values are positioned in different parts of the space.

**Figure 3:**
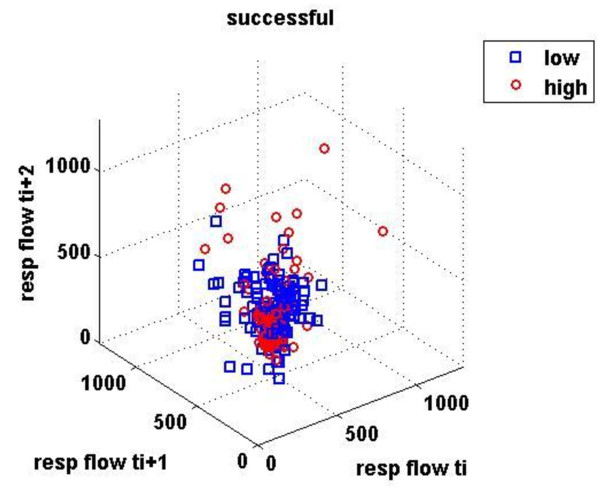
**Phase space of mean inspiratory flow in a weaning success patient**. Different values x(i) of ventilatory flow were plotted against the following ones in tree-dimensional axes: x, x+t, x+2t in Matlab, giving rise to the phase space. Red dots represent data during high PS ventilation, whereas blue dots represent data during the performance of a SBT.

**Figure 4:**
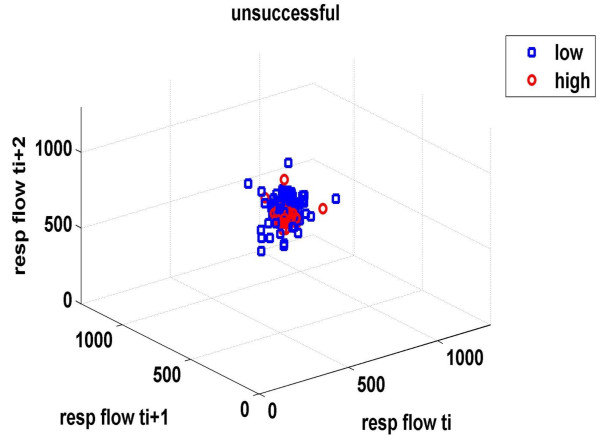
**Phase space of mean inspiratory flow in a weaning failure patient**. Red dots represent data during high PS ventilation, whereas blue dots represent data during the performance of a SBT. Scattering of data seems reduced, compared with findings in figure 3, reflecting decreased variability of the measured parameter. Moreover, flow values are positioned in different parts of the space.

**Figure 5:**
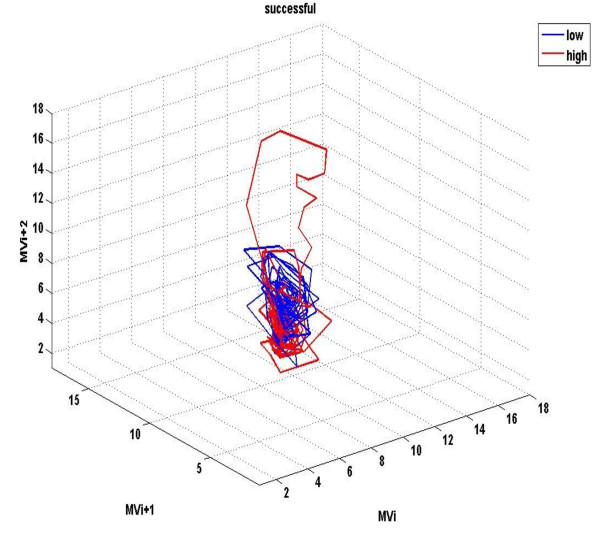
**Phase space of minute ventilation in a weaning success patient**. Different values x(i) of minute ventilation (MV) were plotted against the following ones in tree-dimensional axes: x, x+t, x+2t in Matlab, giving rise to the phase space. Red dots represent data during high PS ventilation, whereas blue dots represent data during the performance of a SBT.

**Figure 6:**
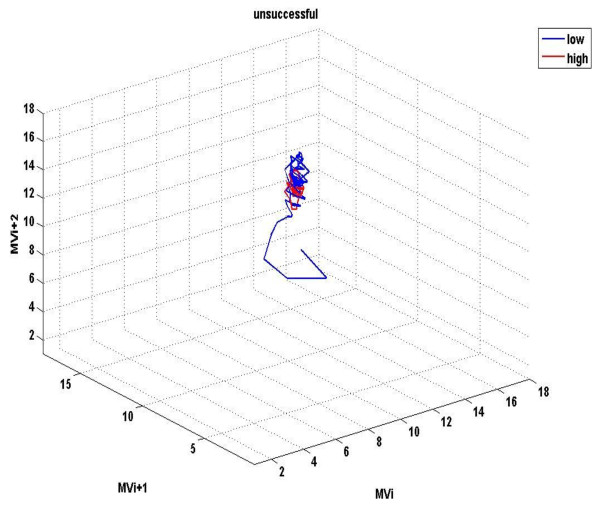
**Phase space of minute ventilation in a weaning failure patient**. Red dots represent data during high PS ventilation, whereas blue dots represent data during the performance of a SBT. Scattering of data seem highly reduced, compared with findings in figure 5, whereas ventilation values are positioned in different parts of the space.

Figures 1 &2 demonstrate the three-dimensional distribution of MV, RR and V_T _in a weaning success and failure patient, respectively. Dispersion of data seems decreased in the second compared with the first graph, reflecting reduced variability of measured parameters. Similarly, figures 3-6 represent the phase space of flow and minute ventilation in two patients with different weaning outcome. In the weaning failure subject (figures 4 and 6) scattering of data seems reduced with limited number of trajectories, indicating relatively simple geometric patterns with more regular shapes.

**Figure 7:**
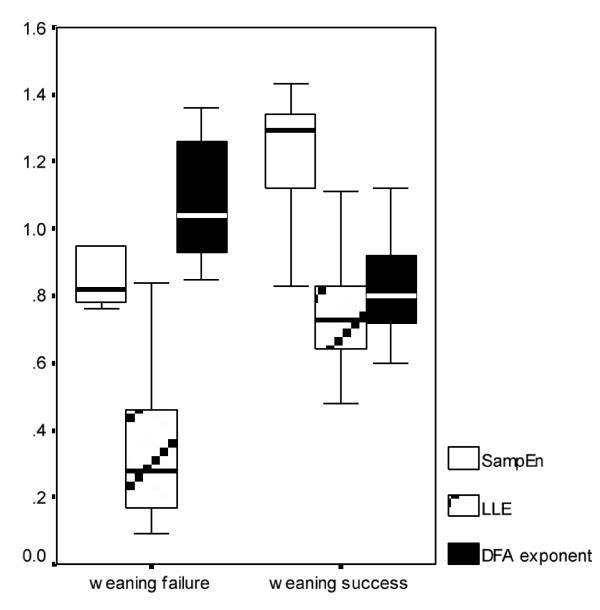
**Box plot of different measured complexity indices**. Box plot of sample entropy (SampEn), DFA and largest lyapunov (LLE) exponents' mean values of mean inspiratory flow time series from patients with different weaning outcome. These metrics were found to differ significantly between weaning success and failure subjects.

DFA exponent of inspiratory flow exhibited inverse changes between and within groups. It increased between the 2 weaning phases (after performing an SBT) in group U [1.08 (0.94-1.25) vs 0.75 (0.63-1.05), p < 0.001] and decreased in group S [0.81 (0.72-0.95) vs 0.94 (0.75-1.12), p < 0.05], whereas at the end of the SBT, group S showed significantly decreased DFA exponent compared with group U (0.81 vs 1.08, p < 0.001). Same trends were also observed for the tidal volume time series but did not reach statistical significance (Table 2, Figure 7).

**Table 2:** Differences of complexity indices between patient subgroups

Parameter	Median	95% CI	Range	p value
	(SE)	(Lower-upper bounds)	(10-90% percentiles)	
Sample entropy of flow				
S	1.26 (0.035)	1.16-1.32	0.87-1.46	< 0.001
U	0.88 (0.027)	0.77-0.94	0.65-1.21	
Sample entropy of V_T_				
S	1.27 (0.075)	0.89-1.47	0.86-1.44	< 0.001
U	0.79 (0.029)	0.54-1.13	0.64-1.92	
DFA exponent of flow				
S	0.81 (0.034)	0.43-1.35	0.72-0.95	< 0.001
U	1.08 (0.05)	0.76-1.13	0.94-1.25	
Largest Lyapunov exponent of flow				
S	0.76 (0.045)	0.56-1.22	0.48-1.11	< 0.001
U	0.27 (0.014)	0.14-0.55	0.09-0.42	

Statistical significant differences of various complexity indices derived from mean inspiratory flow and tidal volume (V_T_) time series analysis, between weaning success (S, n = 22) and weaning failure (U, n = 10) patients. Variables are those that correspond to metrics at the end of the SBT and are presented as median with standard error (SE), along with 95% confidence intervals (CI).

Inverse changes were found in SampEn values of both studied respiratory signals, compared to DFA findings, between and within groups. Thus, decreased entropy values of the inspiratory flow time series were exhibited in weaning failure patients after transition from high to low pressure support ventilation [0.88 (0.65-1.21) vs 1.18 (0.85-1.37), p < 0.001] and increased values in those who separated successfully from the ventilator [1.26 (0.87-1.46) vs 0.82 (0.7-1.1), p < 0.001]. Accordingly, patients from group U manifested reduced entropy values at the end of the SBT compared with those from group S (0.88 vs 1.26, p < 0.001). Entropy values of tidal volume were also found to change similarly with those of respiratory flow [0.79 (0.64-0.92) vs 1.34 (0.88-1.65) for group U and 1.27 (0.86-1.44) vs 0.78 (0.65-0.92) for group S, p < 0.001, respectively, Table 2, Figure 7].

Fractal dimension of both ventilatory signals did not differ significantly between and within groups; however, its values were found to increase at the end of SBT in group S, compared with group U [1.33 (1.25-1.47) vs 1.31 (1.20-1.45) for flow and 1.40 (1.3-1.52) vs 1.38 (1.22-1.49) for V_T_].

Finally, the same trends of change were observed in largest lyapunov exponents of both studied respiratory signals in weaning success and failure patients. LLE of inspiratory flow increased in group S from phase H to phase L [0.76 (0.48-1.11) vs 0.43 (0.23-0.68), p < 0.001] and decreased respectively in group U [0.27 (0.09-0.42) vs 0.65 (0.34-1.04), p < 0.001]. Similar changes but without statistical significance were found in tidal volume signals (Table 2, Figure 7).

Conventional weaning predictors exhibited also significant differences between groups S and U. RSBI, P_0.1 _and their product RSBI* P_0.1 _were significantly increased in subjects who failed a SBT [112.2 (85-143) vs 97.8 (73-116), 1.73 (1.45-1.98) vs 1.48 (1.32-1.86) and 175.25 (134.43-210.3) vs 102.32 (97.84-145.2) respectively, p < 0.005 for all comparisons].

**Figure 8:**
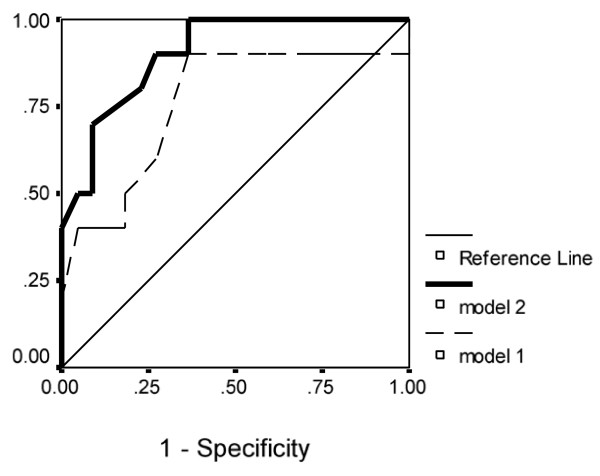
**Receiver operating characteristic curves (ROC) of the two predictive models**. Receiver operating characteristic curves (ROC) of the two models, which were tested for accurately predicting weaning outcome. Model 2 (including RSBI, RSBI* P_0.1_, SampEn and LLE of mean inspiratory flow) performed better, compared with model 1 of conventional indices (RSBI, P_0.1 _and RSBI* P_0.1_).

Stepwise multiple regression analysis demonstrated that RSBI and its product with P_0.1 _were the only conventional variables from model 1 that predicted successfully weaning outcome. Moreover, the combination of RSBI, RSBI* P_0.1_, SampEn and LLE of inspiratory flow (model 2) was found to be more accurate compared to model 1 [R^2 ^= 0.874 with standard error (SE) = 0.215 versus 0.643 with SE = 0.332, p < 0.001, respectively]. The same variables of model 2 according to regression analysis were also selected from the cross-validation analysis as the most accurate and robust predictors of outcome of interest, compared with RSBI and RSBI* P_0.1 _with significantly different values of AUCs (Table 3, Figure 8).

**Table 3:** Cross-validation and ROC curve analysis of the two predictive models

Models	AUC (SE)	Threshold	AUC	Specificity	Sensitivity
			95% CI		
					
**Model 1**	0.831 (0.14)	0.402	0.73-0.92	0.895	0.858
(RSBI, P_0.1 _and RSBI* P_0.1_)					
**Model 2**	0.916 (0.006)	0.296	0.76-0.98	0.967	0.886
(RSBI, RSBI* P_0.1_, SampEn, LLE)					

Mean values of areas under the curve (AUC) with standard errors (SE), 95% confidence intervals (CI) and best threshold among regression coefficients that managed to classify groups with different weaning outcome with the best combined sensitivity and specificity, were computed in Matlab. Three indices (RSBI, P_0.1 _and RSBI* P_0.1_) were included in the model 1 and four indices (RSBI, RSBI* P_0.1, _SampEn and largest lyapunov exponents of mean inspiratory flow time series) were selected in model 2 respectively, which was proven to discriminate more accurately patients with different weaning outcome.

## Discussion

A considerable body of data suggests that healthy individuals exhibit breath-to-breath variability of breath components in a breath series [23,24]. Breath-to-breath variations have been traditionally treated as random uncorrelated white noise superimposed on the output of the respiratory controller [24,25]. According to Tobin, the random fraction aids respiratory system to perform tasks other than gas exchange, such as speaking [26]. Only simple statistics such as mean, variance and coefficient of variation can estimate random variational fraction after averaging over many breathing cycles. Since variability in complex living systems is not only an artefact of biological noise but also an intrinsic property of various control mechanisms, different types of deterministic (non-random) variability have been described in the pattern of breathing [27-29].

Neurons in the brain stem govern respiratory rhythm through a network of coupled oscillators. Critical components of this network are located in a specialised region of the brain stem called the pre-Botzinger complex (pre-BotC) [30]. Del Negro and colleagues showed that progressively elevating neuronal excitability of the pre-BotC of neonatal rats in vitro causes periodic modulation of the inspiratory rhythm, characterised by periodic oscillations, quasiperiodicity and ultimately disorganised aperiodic activity [31]. In another experimental study with anesthetised adult cat models, Chen et al found that both focal hypoxia and chemical stimulation of pre-BotC can produce a marked excitation of phasic phrenic nerve discharge, characterized by reduced complexity, estimated with approximate entropy (low ApEn values) [32]. The above studies support the hypothesis that central respiratory centers are responsible for different breathing patterns with various degrees of variability and complexity in different settings and levels of stimulation. In addition, they can also adapt ventilation to metabolic needs through integration of afferent information.

Apart from chemoreceptor signalling, chest wall and pulmonary receptors may continuously affect central neural output, especially during resistive breathing [33,34]. Brack and Tobin measured breathing variability using CVs and autocorrelation analysis, over one hour in ten patients with restrictive lung disease and in seven healthy subjects. They found that variability of inspiratory time (Ti), expiratory time (Te) and V_T_, were significantly reduced in the patients group compared with the healthy group [26].

Several approaches have been used for the study of respiratory complexity. Donaldson in 8 adults during resting breathing found that different respiratory parameters were characterized by positive lyapunov exponents [35]. Increased respiratory approximate entropy and lyapunov exponents have been reported in patients with panic disorders [36], whereas Akey and colleagues described a reduction in respiratory ApEn upon a hypoxic insult to the brain [37]. Peng who introduced the DFA algorithm for the study of noisy and nonstationary biological signals found that breathing intervals' DFA exponents were significantly decreased in elderly compared to young adults [38]. Finally, hypercapnia has been found to decrease complexity but increase largest lyapunov exponents of different respiratory time series [39].

Implementation of different mathematical tools derived from signal processing techniques for analyzing heart rate and respiratory pattern variability has been shown to provide prognostic information in the assessment of weaning readiness [40,7-10]. Engoren studied 10 control patients who had undergone cardiac surgery and 21 patients who required prolonged (> 7 days) ventilatory support and found increased ApEn values of tidal volume in weaning failure subjects [7]. However and according to a study from Caminal, an inverse relation between the level of pressure support and the CVs of different ventilatory parameters has been established, supporting the view that unloading of respiratory muscles is associated with increased breathing pattern variability, at least in weaning success patients [41]. El-Khatib assessed 52 patients with various disorders during a continuous positive airway pressure (CPAP) trial of 5 cm H_2_O for 60 minutes and found that the CVs, the Kolmogorov-Sinai entropy (sum of largest lyapunov exponents) and the correlation dimension (measure of fractal-like properties) of tidal volume and airway flow were all significantly smaller in the successfully weaning group compared with the failure weaning group [8]. On the contrary, Bien and Wysocki reported decreased variability of different ventilatory parameters in weaning failure patients [9,10]. However, they did not perform non-linear analysis of respiratory time series for assessing breathing complexity. In another interesting study, Vallverdu and colleagues examined heart rate and respiratory pattern complexity in 78 patients during weaning trials using information flow analysis, which describes the regularity of signals by estimating the auto- and mutual information functions. The authors were able to find reduced complexity and a more coupled nonlinear oscillator behavior in weaning failure subjects [42].

To our knowledge, this is the first study in medical literature that applied a 'toolkit' of nonlinear methods in respiratory signals for estimating weaning outcome, in a cohort of surgical patients. In a recently published study that included a similar group of patients and implemented the same weaning protocol, we found reduced complexity and coupling of heart and respiratory rate signals derived from bedside monitors and estimated with DFA and different entropy metrics, in subjects who failed to separate from the ventilator [43]. However, since description of ventilatory complexity requires the assessment of both predictability (entropy) and sensitivity to initial conditions (Lyapunov exponents) of continuous oscillatory signals (flow) and time series of discrete values [24], we decided to apply these methods to different respiratory signals derived from the ventilator. In addition, we tried to assess their scale-invariant properties by computing the fractal dimension. Finally, we applied the DFA algorithm for quantifying fractal properties of respiratory signals through the estimation of long-range correlations, which contrary to fractal dimension, is more suitable for analyzing non-stationary short time series. In conclusion, we assumed that the implementation of different methods for the mathematical description of respiratory complex dynamics that was not performed in our previous report (except for sample entropy), could add significant value in such analysis, in case of significant differences between patients with different outcome of interest. We used the same weaning protocol as Bien, who examined a sample of 78 patients with systemic inflammatory response syndrome (SIRS) [9].

All indices of respiratory complexity were found to exhibit inverse changes between weaning failure and success groups. After the performance of a SBT, increased unloading of the respiratory system was associated with increased breathing complexity in subjects who managed to liberate from the ventilator (figures 1-6), since lower values of DFA exponent and higher values of LLE and SampEn of inspiratory flow were found (figure 7). These results indicate reduced long-range correlations, increased sensitivity to initial conditions and augmented irregularity of flow. Tidal volume signals exhibited similar changes in DFA and LLE without reaching statistical significance, whereas fractal dimension of both signals increased insignificantly in the weaning success group. In conclusion, our findings support the hypothesis that increased ventilatory randomness was associated with weaning success and proved to be more reliable in discriminating patients with different weaning outcome in relation with conventional indices (figure 8). Moreover, ventilatory complexity must be estimated with a combination of nonlinear techniques, since respiratory time series are often very noisy and highly nonstationary, compared with cardiovascular signals [38,43].

These results parallel those from Schmidt and colleagues who reported increased LLE and Kolmogorov-Sinai entropy values of mean inspiratory flow signals in mechanically ventilated patients, after switching the ventilator from the pressure support mode to neurally adjusted ventilatory assist mode (NAVA) [44]. According to these authors, successful spontaneous breathing trials unmask underlying variability and complexity of central neural output, since inspiratory pressure inhibits the respiratory drive. This effect is nicely reflected through the increased complexity indices of flow and is responsible for better neuro-mechanical coupling.

In another study, Mangin and colleagues investigated ventilatory chaotic dynamics in 17 mechanically ventilated patients during switching the ventilator from the assist-control mode to pressure support mode [45]. They were able to show that both fractal dimension and LLE were increased, particularly in 5 patients who were successfully extubated. Furthermore, the authors supposed that increased breathing complexity may also be attributed to higher vagal afferent feedback during unassisted breathing, as has already been shown by Sammon and Bruce [46].

These studies support our findings that transition between mechanical and spontaneous ventilation is associated with increased complexity of respiratory signals in weaning success patients, since duration of ventilation before the SBTs was similar between groups with different weaning outcome. Moreover, in a study of Burykin and Buchman investigating cardiorespiratory dynamics and synchronization during controlled and unassisted breathing in 13 surgical patients, it was demonstrated that mechanical ventilation reduces significantly both heart and respiratory rate complexity whereas spontaneous respiration is more irregular with increased uncoupling of cardiorespiratory rhythms in weaning success patients [47].

Higher variability and complexity of breathing pattern during controlled ventilatory support has been found to ameliorate oxygenation. In an oleic acid injury animal model, Mutch introduced fluctuations according to an algorithm, to mechanical respiration and found increased respiratory arrhythmia and oxygenation and decreased dead space compared with conventional ventilation (with similar MV) [48]. According to Suki, when fluctuations in the form of symmetrically distributed random noise is added to peak airway pressures, the mean does not change but isolated values can be augmented, leading to significant alveolar recruitment [49]. Moreover, low respiratory variability during both controlled and unassisted breathing could deteriorate respiratory mechanics by promoting microatelectasis [10].

Reduced respiratory complexity of flow signals in group U related to group S and between the two phases of PS within weaning failure patients might also reflect loss of effective control mechanisms that govern respiratory rhythms through a network of coupled oscillators [50]. It seems that increased respiratory load reduces complexity of central oscillator output, as it has been suggested from the different studies discussed so far. Moreover, Preas in a clinical study estimating endotoxin effect upon respiratory variability and complexity found a decrease in RR random variability in patients with restrictive lung diseases, a similar pattern of change with Tobin's study [26] and attributed dyspnoea to the endotoxin effect upon brain stem neurons [51]. In our study, the majority of weaning failure patients exhibited dyspnoea and rapid shallow breathing, whereas most of them had high APACHE II scores upon admission. Their characteristics parallel those of the Bien's study, implying the possible presence of SIRS during the weaning trials.

Furthermore, we assume that the observed significant results in different complexity properties for inspiratory flow and not for tidal volume in weaning failure patients could also be attributed to the decreased random variability of RR, which was found in Preas and Tobin's studies. According to Bruce, any respiratory activity includes variability of different types, such as random correlated and uncorrelated, periodic and nonlinear deterministic [24]. Alterations in the random fraction, although not measured in our study, could affect the reliable mathematical description of the non-random one, something that might limit the relevance of these results [24].

For that reason, different techniques, such as the noise titration method, have been implemented for detecting chaotic dynamics [45,52]. However, in this study we did not adopt this technique, since our aim was not to detect the existence of ventilatory chaos but to investigate possible alterations in respiratory dynamics after a specific intervention (SBT). Moreover, we recruited a homogeneous group of patients and it is reasonable to assume that there were no intersubject variations at the level of noise.

Our study suffers several other limitations due to small sample size, which could increase false negative results and be responsible for lack of statistical significance in different non-linear properties of ventilatory signals. In addition, implementation of sophisticated mathematical techniques remains a challenge for average physicians, whereas their standardization is urgently needed, since there is a lack of guidelines for parameter choice and bias to low values, in some cases. Concerning methodological issues, non-linear characterization methods are extremely sensitive to noise and biased when applied to short data sets [24,28]. Another important issue concerns non-filtering of signals, since it has been proven that using filters can distort the characterization of non-linearities [53]. Finally, using low sampling frequency avoids introduction of linearities within the time series, which has been found to occur during oversampling [53].

In conclusion and despite the fact that inspiratory flow and V_T _time series preserved their fractal-like properties, weaning failure patients exhibited reduced breathing pattern complexity during weaning trials, compared with subjects who were successfully separated from the ventilator. Increased respiratory load due to unresolved inflammatory response could be responsible for reducing effective neuro-mechanical coupling. The fact that multiplying the methods and studied parameters did not increase the chance of getting significant results for both flow and tidal volume could be associated with inherent limitations of such methods applied to short and highly noisy time series, patient characteristics or possible presence of unknown covariates. For that reason and based on findings form this and our previous study, we believe that a multimodal monitoring using in addition, both cardiovascular and electroencephalographic signals might increase diagnostic accuracy of such approach. Furthermore, the implementation of other methods such as information flow could be more suitable for studying the highly noisy and nonstationary ventilatory signals. Finally, a comparison between nonlinear properties of heart and different respiratory time series for predicting weaning outcome could shed more light into complex cardiorespiratory interactions during weaning trials.

## Conclusions

Complexity analysis must incorporate many methods that capture different properties of respiratory dynamics. However, we suggest that non-linear analysis of respiratory time during weaning trials might suffer some limitations despite increased diagnostic accuracy compared with conventional weaning indices. A multimodal monitoring of different biosignals derived from both the cardiovascular and respiratory system could increase the value of such methods. Sequential characterization of complex system's complexity could also provide a monitoring tool during weaning trials, at least in surgical patients. The perspective of adopting such techniques as descriptors of the effects of an intervention (SBT) may enhance effectiveness of early extubation. Nevertheless, these findings cannot yield information about weaning prediction in different groups of patients. More studies are needed for the estimation of their value in other sub-group categories, and for the quantitative assessment of changes during different weaning protocols.

## List of abbreviations

**APACHE**: Acute Physiology and Chronic Health Evaluation; **ApEn**: approximate entropy; **AUC**: area under the curve; **DFA**: detrended fluctuation analysis; **COPD**: chronic obstructive pulmonary disease; **CPAP**: continuous positive airway pressure; **CV**: coefficient of variation; **FD**: fractal dimension; **FW**: failure weaning; **HR**: heart rate; **ICU**: intensive care unit; **LLE**: lyapunov exponents; **MV**: minute ventilation; **PEEP**: positive end-expiratory pressure; **P**_**0.1**_: airway occlusion pressure at 0.1 sec; **PSV**: pressure support ventilation; **ROC**: receiver operating characteristic; **RR**: respiratory rate; **RSBI**: rapid shallow breathing index; **SampEn**: sample entropy; **SBT**: spontaneous breathing trial; **SIMV**: synchronized intermittent mandatory ventilation; **SIRS**: systemic inflammatory response syndrome; **SW**: successful weaning; **V**_**T**_: tidal volume.

## Competing interests

The authors declare that they have no competing interests.

## Authors' contributions

VEP was the principal investigator who designed the study, collected data, helped with data analysis and wrote the manuscript. IGC was responsible for data analysis, NKM reviewed, edited and finally approved methods of data analysis, IAP supervised the whole study. All authors have read and approved the final manuscript.
